# Ultrastructural changes in endothelial cells of buffaloes following in-vitro exposure to *Pasteurella multocida* B:2

**DOI:** 10.1186/s12917-020-02415-2

**Published:** 2020-06-09

**Authors:** Yulianna Puspitasari, Annas Salleh, Mohd Zamri-Saad

**Affiliations:** 1 Research Centre for Ruminant Diseases, Faculty of Veterinary Medicine, Universiti Putra Malaysia, 43400 Serdang, Malaysia; 2 Department of Veterinary Microbiology, Faculty of Veterinary Medicine, Universitas Airlangga, East Java, 60115 Indonesia

**Keywords:** Buffalo, Endothelial cells, *Pasteurella multocida*, Endotoxin

## Abstract

**Background:**

*Pasteurella multocida* B:2 causes haemorrhagic septicaemia in cattle and buffaloes. However, buffaloes are found to be more susceptible to the infection than cattle. Upon infection, the pathogen rapidly spread from the respiratory tract to the blood circulation within 16-72 h, causing septicaemia. So far, limited study has been conducted to evaluate the response of endothelial cells of buffalo towards *P. multocida* B:2 and its lipopolysaccharide (LPS). This study aimed to evaluate the ultrastructural changes in the aortic endothelium of buffaloes (BAEC) following exposure to *P. multocida* B:2 and its endotoxin. The endothelial cells were harvested from the aorta of healthy buffaloes and were prepared as monolayer cell cultures. The cultures were divided into 3 groups before Group 1 was inoculated with 10^7^ cfu/ml of whole cell *P. multocida* B:2, Group 2 with LPS, which was extracted earlier from 10^7^ cfu/ml of *P. multocida* B:2 and Group 3 with sterile cell culture medium. The cells were harvested at 0, 6, 12, 18, 24, 36, and 48 h post-inoculation for assessment of cellular changes using transmission electron microscopy.

**Results:**

The BAEC of Groups 1 and 2 demonstrated moderate to severe endothelial lysis, suggestive of acute cellular injury. In general, severity of the ultrastructural changes increased with the time of incubation but no significant difference (*p* > 0.05) in the severity of the cellular changes between Groups 1 and 2 was observed in the first 18 h. The severity of lesions became significant (*p* < 0.05) thereafter. Both treated Groups 1 and 2 showed significantly (p < 0.05) more severe cellular changes compared to the control Group 3 from 6 h post-inoculation. The severity reached peak at the end of the study period with score 3 for Group 1 and score 2.8 for Group 2.

**Conclusions:**

This study revealed that both whole cells *P. multocida* B:2 and LPS endotoxin showed similar moderate to severe cellular damage, but whole-cell *P. multocida* B:2 appeared to be more potent in causing much severe damage than LPS alone.

## Background

*Pasteurella multocida* B:2 is a Gram-negative bacterium, known to cause haemorrhagic septicaemia (HS) in cattle and buffaloes [1]. It was previously found that buffaloes are more susceptible to the infection compared to cattle [2], thus higher rate of mortality was reported among buffaloes [3]. Pathogenesis of the disease involves entry of the pathogen via intranasal or oral route [4, 5] leading to rapid infection of the lungs. For septicaemia to develop, the pathogen needs to cross the endothelial cell and other associated structures [6]. This translocation of the pathogen across the capillary causes severe lysis of cattle endothelium [7].

Endothelial injuries have been observed in the early events following infection by *P. multocida* and the closely-related bacterial species, the *Mannhaeimia haemolytica* [8]. A previous ultrastructural study postulated that the interaction between *P. multocida* B:2 and the host endothelial cells could lead to severe damage and destruction that enables *P. multocida* B:2 to translocate from blood vessels into the organs and vice versa [9]. In-vitro studies revealed that *P. multocida* B:2 has the ability to adhere and invade the bovine aortic endothelial cells and embryonic bovine lung cells [7, 10].

There is no study, however, on the response by endothelial cells of buffaloes to in-vitro infection with live *P. multocida* and the endotoxin LPS. Since buffalo is more susceptible to HS, a study using buffalo would be beneficial in evaluating the ultrastructural changes, especially pertaining to the severity of endothelial cell damage. Moreover, different strains of *P. multocida* isolated from different hosts with different diseases are known to have different degree of invasiveness [10]. Considering this paucity of information and high susceptibility of buffaloes to *P. multocida* B:2, this experiment was conducted to evaluate the in-vitro damages on the endothelial cells of buffalo following exposure to whole cell and endotoxin of *P. multocida* B:2.

## Results

### Ultrastructural changes in buffalo aortic endothelial cells (BAEC) following exposure to *P. multocida* B:2

Following inoculation with *P. multocida* B:2, the BAEC monolayer revealed ultrastructural lesions suggestive of acute cellular injury with moderate to severe score. Generally, there was an increasing pattern of lesion scores with time of incubation. The score significantly (*p* < 0.05) increased in the first 24 h but remained insignificant (*p* > 0.05) thereafter.

At 0 h, there were mild dilatation of the rough endoplasmic reticulum (RER) with few mitochodria showing mild swelling. Cytoplasmic vacuolation and blebbing were also observed. At 6 h, lesions in RER and other cytocavity network were more severe leading to frequent vesiculations. Mitochondrial swelling was occasionally accompanied by partial cristolysis. Cytoplasmic blebs were noted with prominent nuclear invagination and nuclear chromatin peripheralisation. Large numbers of *P. multocida* B:2 were observed outside the cell membrane of BAEC. The lesion score was significantly (*p* < 0.05) higher compared to 0 h p.i.

**Fig. 1:**
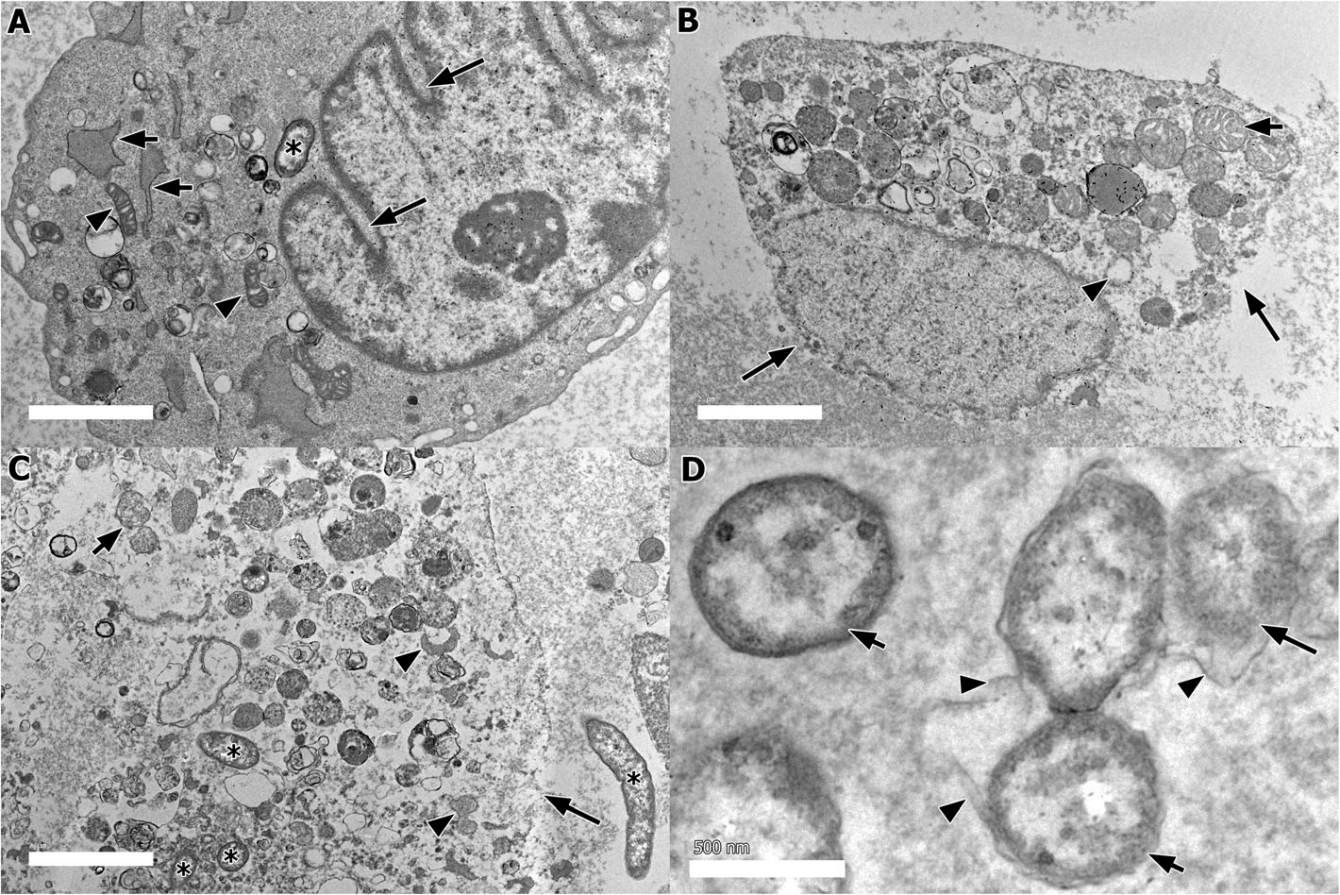
Ultrastructural changes in BAEC exposed to *P. multocida* B:2. **a** Severe nuclear invagination (long arrow), with dilatation, fragmentation, and vesiculation of RER (short arrows). Few mitochondria had cristae disarrangement and partial cristolysis (arrowhead), with low number of *P. multocida* B:2 (asterisk) in the cytoplasm. (bar = 2 μm, TEM). **b** Disintegration of plasma membrane and nuclear envelope (long arrows), with prominent swollen mitochondria and cristae disarrangement (short arrow) and RER vesiculation (arrow head) (bar = 2 μm, TEM). **c** Presence of numerous *P. multocida* B:2 (asterisks) inside and outside of a lysed BAEC (long arrow). Mitochondria cristolysis (short arrow) and ribosomal degranulation from RER membranes with accumulation of ribosomal fragments in the cytosol were also noted (arrow heads) (bar = 2 μm, TEM). **d**. Presence of lysed (long arrow) and intact (short arrows) *P. multocida* B:2 with shedding of outer membrane (arrowheads). (bar = 500 nm, TEM)

At 12 h, there were extensively dilated cisternae with fragmented and vesiculated RER, forming large vacuoles (Fig. 1a). Some mitochondria were swollen with disarranged cristae and partial cristolysis was occasionally observed. Cytoplasmic blebs were also seen while the nuclear membranes were observed with severe nuclear invagination (Fig. 1a) along with peripheral nuclear condensation. Few *P. multocida* B:2 were found localized within the cytoplasm despite the plasma membrane remained intact. The lesion score was significantly (*p* < 0.05) higher compared to 6 h p.i.

At 18 h, much severe ultrastructural lesions were observed. The plasma membrane started to show discontinuity and disintegration, leading to endothelial lysis while the nuclear matrix was rarefied (Fig. 1b). Multifocal vesiculations were evident, affecting the membrane of RER. Most mitochondria appeared swollen, often with partial cristolysis. Numerous cells of *P. multocida* B:2 were found within the cytoplasm (Fig. 1c). Compared with the previous incubation time, the lesion score at 18 h post-incubation was significantly (*p* < 0.05) higher. At 24 h p.i., most BAEC had lysed, releasing the intracellular contents, while the remaining BAEC showed disintegration of the cell membrane but the cytoplasmic organelles remained inside of the cell. Numerous *P. multocida* B:2 cells were seen adjacent to the remnants of the lysed cells. Some of the bacterial cells had lysed, shedding the outer membrane (Fig. 1d). The ultrastructural lesion score was significantly (*p* < 0.05) higher compare to 18 h p.i.

The lesion scores at 36 h and 48 h p.i. were similar, while all BAEC were completely lysed, leaving the cellular remnants intermixed with numerous cells of *P. multocida* B:2. No significant (*p* > 0.05) difference in lesion scores was observed between 24 h, 36 h and 48 h p.i.

### Ultrastructural changes in BAEC following exposure to LPS endotoxin of *P.multocida* B:2

Ultrastructural examination of Group 2 revealed similar moderate to severe lesion scores suggestive of acute cellular injury. In general, there was increasing pattern of lesion scores with time of incubation. The lesion scores showed significant increasing pattern with time (*p* < 0.05) in the first 12 h p.i. before the scores became insignificant (*p* > 0.05) thereafter.

At 0 h, mild celullar changes were noted with few RER were dilated and some mitochondria were mildly swollen. Cytoplasmic vacuolation and blebbing were also occasionally observed. At 6 h, some RER had dilated cisternae, leading to fragmentation, and vesiculation. There was ribosomal degranulation from RER membranes with accumulation of ribosomal fragments in the cytosol. Mitochondrial swellings were frequently observed. The nucleus showed severe invagination with chromatin peripheralisation while the cytoplasm had blebs and vacuolations. The lesion score was significantly (*p* < 0.05) higher compared to 0 h.

**Fig. 2:**
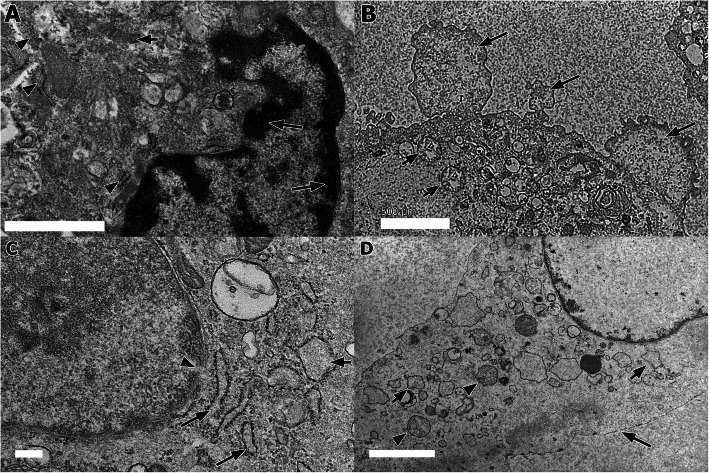
Ultrastructural changes in the BAEC following exposure to LPS endotoxin of *P. multocida* B:2. **a** Nuclear changes exhibited by peripheral nuclear condensation (long arrows) with mitochondrial swelling (short arrow) and dilated, fragmentation and vesiculation of RER membrane (arrowheads) (bar = 1 μm, TEM). **b** Blebbing of cytoplasmic membrane (long arrows), and swelling and cristolysis in mitochondria (short arrows) (bar = 500 nm, TEM). **c** Dilatation (long arrows) and vesiculation (short arrow) of RER. Ribosomal degranulation from RER resulted in ribosomal fragments in the cytosol. Note the discontinued nuclear membrane (arrowhead). (bar = 200 nm, TEM). **d** Discontinuation of plasma membrane (long arrow). Noted the RER vesiculation (short arrows) and swelling and cristolysis of mitochondria (arrowheads) and (bar = 2 μm, TEM)

At 12 h p.i., there were extensive dilatation, fragmentation, and vesiculation of RER and other cytocavity network. The mitochondria showed cristae disarrangement with partial cristolysis. The cytoplasms had blebs and vacuolation while the nuclear membranes showed severe invagination along with peripheral nuclear condensation (Fig. 2a). The lesion score was significantly (*p* < 0.05) higher compared to 6 h. Incubation for 18 h resulted in cytoplasmic blebbings (Fig. 2b), severe nuclear invagination and peripheral nuclear condensation. There were numerous and extensively distributed dilation, fragmentation and vesiculation of RER membranes. Severe alterations of the mitochondria with swelling and cristolysis were also observed. When compared with the previous incubation time, the scores were insignificantly (*p* > 0.05) higher than 12 h p.i.

At 24 h, cytoplasmic blebbings were frequently observed. Markedly severe invagination of the nuclear membrane and nuclear chromatin peripheralisation were occasionally accompanied by discontinuation of the nuclear membrane. Most mitochondria exhibited extensive swelling, accompanied by partial to complete mitochondria cristolysis. Dilated cisternae, fragmentation, and vesiculation of RER membranes with ribosomal degranulation were consistently observed (Fig. 2c). The score was higher but not significant (*p* > 0.05) compared with 18 h p.i.

At 36 h and 48 h, there were more BAEC cells that showed extensively discontinued cell membranes (Fig. 2d) while other changes were similar with the previous incubation time. When compare with the previous time of incubation, the scores were not significantly (*p* > 0.05) different.

### Ultrastructural changes in BAEC following introduction of sterile medium

**Fig. 3:**
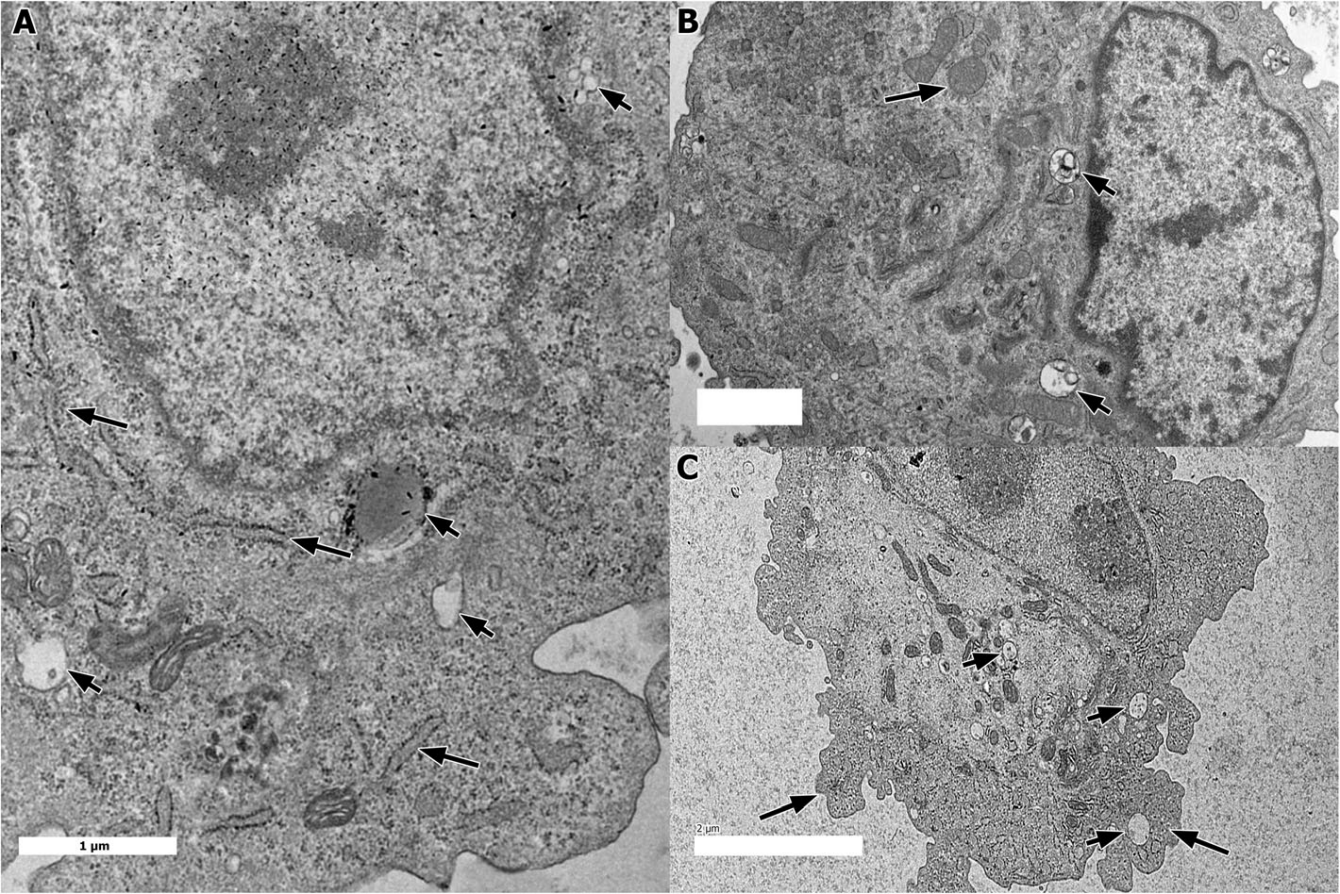
Ultrastructural changes in the BAEC following exposure to sterile medium. **a** Mild RER dilatation (long arrows) and cytoplasmic vacuolation (short arrows) (bar = 1 μm, TEM). **b** Mitochondrial swelling (long arrow) and cytoplasmic vacuolation (short arrows) (bar = 1 μm, TEM). **c** Mild cytoplasmic blebbing (long arrows) and cytoplasmic vacuolation (short arrows) (bar = 2 μm, TEM)

Ultrastructural examination of the BAECs monolayer following in-vitro inoculation with sterile culture medium (Group 3) revealed only mild changes. In general, the scores remained insignificantly (p > 0.05) similar at all times of inoculation. There were mild celullar changes in the form of slight dilatation of RER (Fig. 3a) and mitochodrial swelling (Fig. 3b). Mild cytoplasmic vacuolation and blebs (Fig. 3c) were occasionally observed.

### Comparison of ultrastructural lesion scores of all groups

**Fig. 4:**
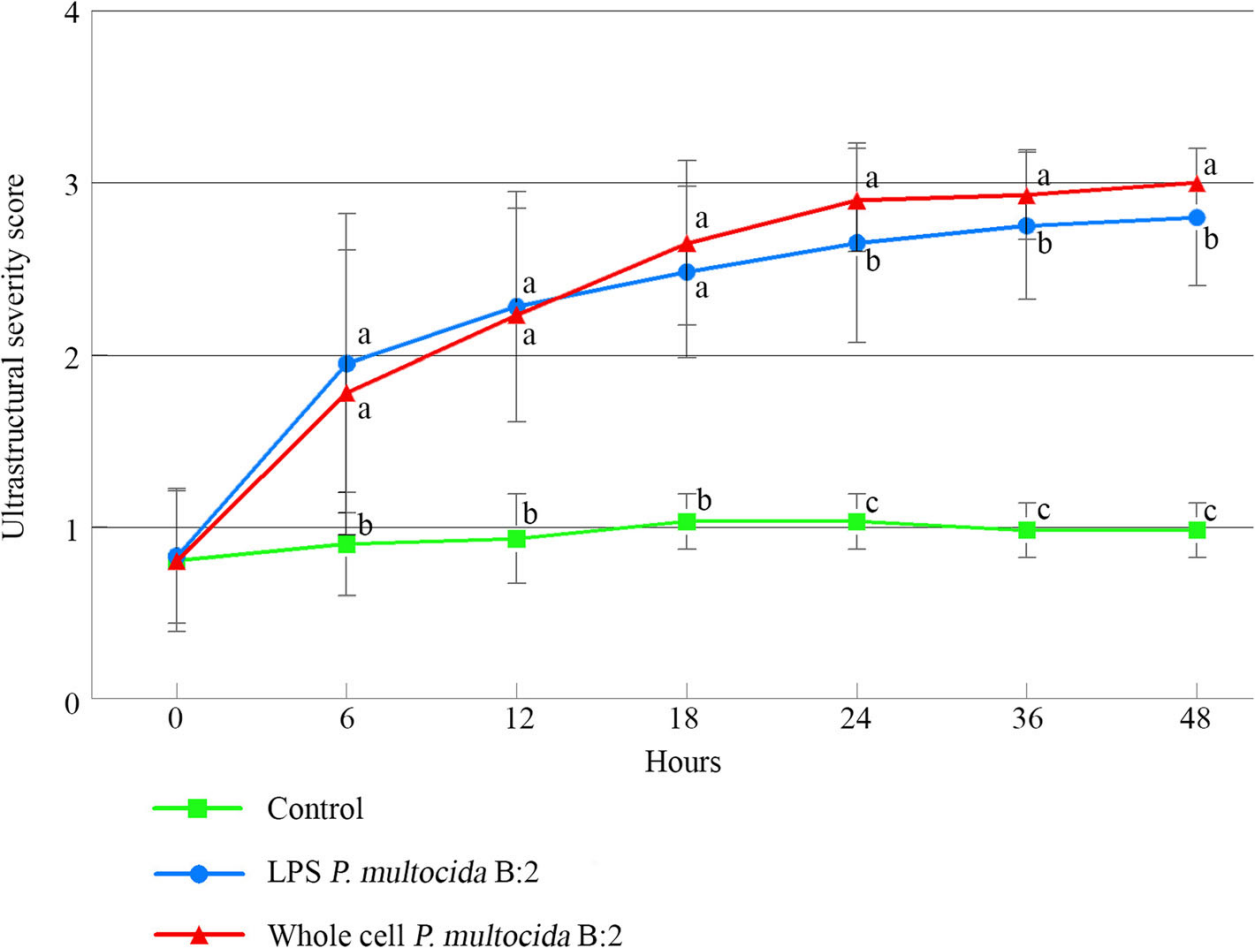
Ultrastructural scoring pattern of BAEC following exposure to whole cell and LPS endotoxin of *P. multocida* B:2 at different times post-inoculation (mean ± SD). Different letters (a,b,c) indicates statistical significance (*p* < 0.05) at each point of time. Total number of buffaloes: 3, number of BAEC culture per sampling per group: 3, number of ultramicroscopic fields per sample: 15, magnification power: × 5000, statistical analysis: one-way ANOVA

Following in-vitro inoculation of whole cell and endotoxin of *P. multocida* B:2, the BAEC cells demonstrated moderate to severe changes suggestive of acute cellular injuries. There was general increased in the lesion scores of both exposed groups with time of incubation (Fig. 4). However, there were no significant differences (*p* > 0.05) in the lesion score between the two exposed groups in the first 18 h p.i. before the lesion scores became significant (*p* < 0.05) thereafter. Otherwise, all exposed groups showed significantly (p < 0.05) higher scores compared to control Group 3 from 6 h onwards.

At 0 h, all groups showed mild cellular changes with scores of 0.80 ± 0.41, 0.83 ± 0.39, 0.88 ± 0.34 for Groups 1, 2 and 3, respectively. The differences were not significant (*p* > 0.05). At 6 h, the score for Group 2 was insignificantly (p > 0.05) higher (1.95 ± 0.87) than Group 1 (1.78 ± 0.83) but were significantly (*p* < 0.05) higher than Group 3 (0.90 ± 0.30). Similarly, at 12 h, the scores were insignificant (p > 0.05) between Groups 1 and 2 with scores of 2.23 ± 0.62 and 2.28 ± 0.67, respectively but were significantly (*p* < 0.05) higher than the control Group 3 (0.93 ± 0.26). At 18 h, Group 1 showed insignificantly (p > 0.05) higher score (2.65 ± 0.48) than Group 2 (2.48 ± 0.50), but significantly (*p* < 0.05) higher than control Group 3 (1.03 ± 0.16).

However, at 24 h, Group 1 (2.90 ± 0.30) was significantly (*p* < 0.05) higher than Groups 2 (2.65 ± 0.58) and 3 (1.03 ± 0.16). Moreover, significantly (p < 0.05) higher score was demonstrated by Group 1 (2.93 ± 0.26) when compared with Group 2 (2.75 ± 0.43) and Group 3 (0.98 ± 0.16) at 36 h. Similarly, at 48 h, lesion score for Group 1 reached peak at 3.00 ± 0.00, which was significantly (p < 0.05) higher than 2.80 ± 0.40 score of Group 2 and 0.98 ± 0.16 score of Group 3. In addition, Group 3 showed significantly (p < 0.05) lowest lesion scores compared to the two exposed groups.

## Discussion

Damages and eventually destruction of the endothelial cells is a mechanism that enables *P. multocida* B:2 to translocate from organs into blood vessels and vice versa, leading septicaemia [6]. The present study describes and evaluate for the first time the severity of ultrastructural lesions shown by the BAEC following exposure to *P. multocida* B:2 and its LPS endotoxin to better understand the pathogenesis of HS.

LPS is a known major component of *P. multocida* that plays an important role in the pathogenesis of HS in buffaloes [11]. Experimental studies have shown that inoculation of the endotoxin would reproduce comparable clinical signs and pathological lesions as the field HS [2, 11]. In this study, exposure of BAEC to both whole cell and endotoxin of *P. multocida* B:2 causes moderate to severe ultrastructural lesions suggestive of acute cellular injury. In most endothelial cells, these eventually lead to endothelial lysis. The BAEC exposed to *P. multocida* B:2 or its LPS showed increasing lesion scores with time of incubation that reached peak at 48 h. Previous in-vivo experimental study concluded that infected buffalo calves showed clinical signs of HS followed by death between 6 and 48 h [9]. Another study concluded that septicaemia could develop between 2.5 and 12 h [2]. The early lysis of BAEC observed in this study following exposure to *P. multocida* B:2 and its endotoxin provide an elaboration of the rapid development of circulatory failure and death of buffalo with HS.

In general, whole cell *P. multocida* B:2 seemed to be slightly more potent in inducing lesions in the endothelium as it caused much severe ultrastructural damages to the BAEC than the LPS alone after 18 h. This could possibly be due to the capability of *P. multocida* B:2 to invade the endothelium and also to release high amount of endotoxin during rapid multiplication. This is supported by the previous findings that Gram-negative bacteria release endotoxin mostly during multiplication and destruction stages [12]. Moreover, it was found that *P. multocida* B:2 tends to attach to and colonize the lung as early as 2 h [13]. After colonisation, *P. multocida* B:2 rapidly multiplied and releases endotoxin in high amount. In fact, the endotoxin could be detected in the blood of infected animals prior to the detection of *P. multocida* B:2 in the blood [2].

Following exposure of BAEC to whole-cell *P. multocida* B:2, the bacterium could be observed intracellularly within both intact and lysed BAEC. This is due to the fact that *P. multocida* has the ability to adhere and invade bovine aortic endothelial cells and the embryonic bovine lung cells [7, 10]. Should similar mechanism of invasion applies to both cattle and buffalo, it could be explained by the fact that LPS destabilized the cells actin filaments, which facilitates the invasion [14]. Observation on the invasion into BAEC suggests that *P. multocida* B:2 could actually inflict injury to the endothelium and pneumocyte from inside and outside of the cells hence, the severe ultrastructural lesions in the cytoplasmic organelles and nucleus of the exposed BAEC.

As consistently observed in the present study, ultrastructural cellular changes eventually resulted in cell lysis, suggesting that *P. multocida* B:2 endotoxin is extremely detrimental to the host endothelial cells. Similarly, in bovine leukocytes that were treated with LPS of *Pasteurella multocida*, it was found that mitochondrial dysfunction was a major event that contributes to the cell death [15]. Binding and internalization of LPS across plasma membrane is known to cause aggregation of LPS on organelles, which then interferes the cell physiology leading to nuclear membrane injury and cell death [16]. In this study, it was observed that mitochondria of BAEC exposed to *P. multocida* B:2 or its LPS exhibited lesions as early as 6–12 h. In fact, it is believed mitochondrial dysfunction is a key early cellular event in development of organ failure in septicaemic diseases or sepsis [17].

Elevated levels of *P. multocida* B:2 LPS could likely reinforced cellular damages of the host cells, leading to acute death. The LPS of *P. multocida* has been shown to activate cell proliferation and cytokine-mediated inflammation at certain threshold level while at higher levels, cell death and tissue pathology are induced [15]. Even though, cellular activation by the LPS of *P. multocida* resulted in cytokine production and protective inflammatory changes, induction of cell death could result in loss of immunocompetent cells that inhibit uncontrolled bacterial replication [18, 19]. Moreover, recent in-vitro study revealed that *P. multocida* B:2 has high ability to cause death of phagocytic cells, particularly in the neutrophil and macrophage following infection with *P. multocida* B:2. This might be attributed by the release of large amount of LPS endotoxin upon intracellular lysis of *P. multocida* B:2 [20]. Crucially, during acute phase of infection, LPS-induced immune cell death could also results in collateral tissue damage, aggravating sepsis-like response [18, 21]. Thus, immuno-pathological changes of acute pasteurellosis could result from a combined effects of bacterial products and host inflammatory factors [18]. Further studies should be conducted to explore the actual and detailed mechanisms of *P. multocida* B:2 LPS-mediated cell death and its contribution in immuno-pathogenesis of acute HS.

## Conclusions

Exposure of BAEC to *P. multocida* B:2 or its endotoxin leads to ultrastructural damages in the BAEC. Invasion of *P. multocida* B:2 into BAEC could induce BAEC damages that lead to cell lysis. The findings highlighted the possible mechanism of translocation of *P. multocida* B:2 across capillaries to produce septicaemia and death.

## Methods

### Animals

Three healthy buffaloes (*Bubalus bubalis*) of 1 year of age were bought from a private farmer. These buffaloes were not previously vaccinated. They were transported to an experimental animal facility, where they were kept in individual pen and fed with cut grass at the rate of 4 kg/buffalo/day. Commercial feed was provided as supplement at the rate of 500 g/buffalo/day, while drinking water was available ad libitum. These buffaloes were ensured to be free from *P. multocida* prior to the study. This was achieved by collection of deep nasal swab samples at weekly interval for 2 weeks [22]. The swab samples were subjected for bacterial isolation and identification using polymerase chain reaction [23].

### Preparation of *P. multocida* B:2 inoculum and purification of lipopolysasccharide endotoxin

Wild-type *P. multocida* B:2 isolated from an outbreak of HS was used in this study. It was prepared to achieve an infective dose of 1.0 × 10^7^ cfu/ml of live *P. multocida* B:2 as previously described [20]. Briefly, it was cultured onto blood agar and incubated at 37 °C for 24 h. Four colonies from this blood agar were transferred into a brain-heart infusion broth and incubated at 37 °C for 18 h with shaking at 150 rpm for. Bacterial concentration was determined using serial dilution method prior to adjustment to the aforementioned infective dose.

The LPS was extracted from the prepared 1.0 × 10^7^ cfu/ml of live *P. multocida* B:2 using chloroform method from an extraction kit (Intron Biotechnology) as previously described [24].

### Isolation and culture of BAEC

**Fig. 5:**
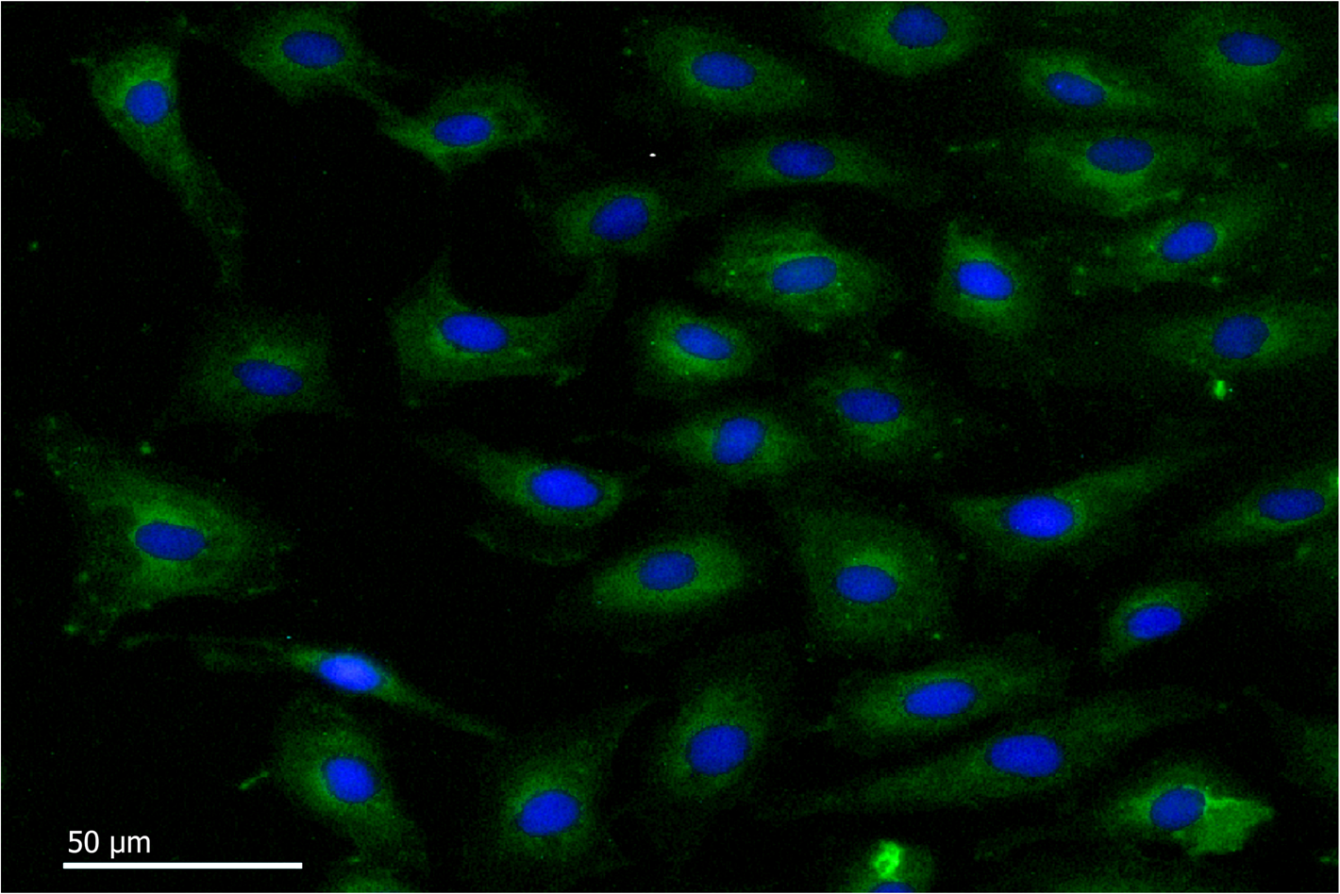
Immunofluorescence characterization of BAEC. BAEC labelled with rabbit anti-bovine factor VIII as primary antibody and Alexa Fluor® 488 goat anti-rabbit IgG H&L as the secondary antibody (For blue stained nuclei, cells labeled with DAPI) (bar = 50 μm)

The buffaloes were euthanized by halal slaughter method [25], followed by immediate collection of the aortas. During collection, both ends of the ascending aorta were clamped, excised, and wrapped. The lumen was then filled with cold DMEM media containing 2% antibiotic-antimycotic (mixture of penicillin, streptomycin, and amphotericin B) (Gibco, USA) [26]. The aortic samples were placed in phosphate-buffer saline (PBS) supplemented with 6% antibiotic-antimycotic (Gibco, USA) [26] and transported to the laboratory in an ice box. At the laboratory, the clamps were removed and the aortas were washed with cold PBS before the adventitia was removed. The aortas were cut into two pieces of 5 cm long before each piece was excised longitudinally. They were then placed in separate dishes and 3 ml of 0.1% collagenase type 1 solution (Worthington Biochemical, US) was dripped onto the endothelial surface. Next, the tissues were incubated at 37 °C with 5% CO_2_ for 30 min. Each aorta was placed in 15 ml tube with 10 ml of sterile DMEM supplemented with 20% foetal bovine serum (FBS), 2 mM L-Glutamine, and 1% antibiotic-antimycotic (Gibco, USA). Then, sterile cotton swabs were moistened with the medium. The swab was touched onto the epithelial surface and was drawn longitudinally with moderate pressure while rotating the swab slowly as it progressed. It was then dipped into the tube of medium and swirled gently to dislodge the harvested cells. The tube was centrifuged at 3000 rpm at 4 °C for 10 min. The supernatant was discarded and the pellet was re-suspended in 5 ml DMEM supplemented with 20% FBS. The cells were then plated in 25 cm^2^ tissue culture flask and incubated at 37 °C with 5% CO_2_ overnight. Cellular debris and unattached cells were washed away with HBSS without Calcium and Magnesium (Gibco®, USA). The isolated cells were established as primary cultures in DMEM with 20% FBS, 2 mM L-Glutamine, and 1% antibiotic-antimycotic (Gibco, USA). The cells were serially passaged and maintained in complete medium. The 4th passage of BAEC were used for experimental assays [10]. At this stage, confirmation of endothelial cells was made through the presence of clotting factor VIII-related antigen by immunofluorescence using anti-factor VIII serum (Abcam, UK) (Fig. 5), as previously described [26] with slight modification. Trypan blue exclusion test [20] was applied to determine the cells’ viability using a standard haemocytometer (Hausser Scientific, USA).

### Experimental design

Approximately 1 × 10^5^ cells/ml of the BAEC were seeded into chamber slides and incubated overnight at 37 °C with 5% CO_2_. Before exposure, the monolayers were washed with HBSS (Gibco®, USA). Then, 10^7^ cfu of live *P. multocida* B:2 inoculum in cell culture medium without antibiotics was inoculated onto the BAEC monolayers at the bacteria:cell ratio of 100:1 and was known as Group 1. BAEC monolayers of Group 2 were similarly exposed to the LPS broth extracts, which was extracted earlier from 10^7^ cfu of live *P. multocida* B:2. Group 3 was similarly inoculated with sterile cell culture medium but with neither *P. multocida* B:2 nor LPS. The cells were harvested at 0, 6, 12, 18, 24, 36 and 48 h post-inoculation (p.i.). At those pre-determined time intervals, 3 monolayers from each group were harvested and processed before the cells were observed under transmission electron microscopic (TEM) to assess the ultrastructural changes.

### Transmission Electron microscopy

The harvested monolayer cells were centrifuged at 500 G for 10 min. The resultant pellet was fixed in 2.5% (v/v) glutaraldehyde in 0.1 M sodium cacodylate buffer (pH 7.2) at 4 °C for 4 h. This was followed by centrifugation at 5000 rpm for 5 min, removal of the fixative and addition of 1 ml of horse serum to submerge the samples. The serum was allowed to clot before the clotted samples were diced into 1 mm^3^ and fixed in 2.5% glutaraldehyde for 2 h at 4 °C, washed three times with 0.1 M sodium cacodylate buffer of 10 min each and post-fixed in 1% osmium tetroxide for 2 h at 4 °C. The samples were then re-washed with 0.1 M sodium cacodylate buffer three times of 10 min each, dehydrated with graded acetone at 35, 50, 75, 95% for 10 min each and 100% for 15 min for 3 changes. The specimens were then infiltrated with a mixture of acetone and resin at a ratio of 1:1 for 1 h, continued at ratio 1:3 for 2 h before being dipped into 100% resin overnight and then in 100% resin for 2 h. Finally, the samples were embedded in beam capsules with resin, before being polymerized in an oven at 60 °C for 48 h. Ultrathin sections were obtained using an ultra-microtome and the sections were stained with 2% (w/v) uranyl acetate (Agar Scientific) and lead citrate (Agar Scientific). The samples were observed under TEM (H-7100; Hitachi, Japan) operating at 100 kV.

### Ultrastructural examination and evaluation

Fifteen random microscopic fields at 5000x magnification of each of the triplicate monolayer cell samples were examined. The ultrastructural changes were described and scored based on the characteristics of ultrastructural responses by the cells [27]. The response to cellular injury was considered as mild if predominant changes were of early events and scored 1, moderate when predominant changes were of intermediate events and scored 2, while changes considered severe were scored 3. Score 0 was given if the section appeared normal (Table 1).

**Table 1:** Scoring and severity of scoring criteria for ultrastructural changes following in-vitro inoculation by whole cell and LPS endotoxin of *P. multocida* B:2

Normal Score 0	Early events (Mild) Score 1	Intermediate events (Moderate) Score 2	Late events (Severe) Score 3
Normal organelles	Mitochondrial swelling	Mitochondrial cristolysis	Mitochondrial vacuoles
	Cytocavity network dilation	Cytocavity network membrane fragmentation	Cytoplasmic vesiculation
	Nuclear envelope dilatation	Nuclear envelope collapse and invagination	Nuclear envelope disintegration
	Eccentric nucleoli	Nucleoplasm rarefaction	Nuclear chromatin peripheralisation

#### Statistical analysis

The data were analysed using one-way ANOVA and LSD multiple comparison to compare the severity in different organelles between whole cells and LPS. The data were expressed as mean of severity and considered significant at *p* < 0.05. All statistical analyses were done using SPSS software, version 22.0.

## Data Availability

The datasets used and/or analysed during the current study are available from the corresponding author on reasonable request.

## Abbreviations

BAEC: buffalo aortic endothelial cell
LPS: lipopolysaccharide
*P. multocida*: *Pasteurella multocida*
HS: haemorrhagic septicaemia
SDS-PAGE: sodium dodecyl sulfate–polyacrylamide gel electrophoresis
RER: rough endoplasmic reticulum
ANOVA: analysis of variance
LSD: least significant difference
